# Phase I/II trial of doxorubicin and fixed dose-rate infusion gemcitabine in advanced soft tissue sarcomas: a GEIS study

**DOI:** 10.1038/sj.bjc.6603187

**Published:** 2006-05-23

**Authors:** A López-Pousa, R Losa, J Martín, J Maurel, J Fra, M Sierra, A Casado, J García del Muro, A Poveda, C Balañá, J Martínez-Trufero, E Esteban, J M Buesa

**Affiliations:** 1 Department of Medical Oncology, Hospital Sant Pau, Avda. Sant Antoni Ma Claret 167, 08025 Barcelona, Spain; 2 Department of Medical Oncology, Hospital Central de Asturias, Instituto Universitario de Oncología del Principado de Asturias (IUOPA) – Obra Social Cajastur. Julián Clavería s/n, 33006 Oviedo, Spain; 3 Department of Medical Oncology, Hospital Son Dureta, Andrea Doria 55, 07014 Palma de Mallorca, Spain; 4 Department of Medical Oncology, Hospital Clínic, Villarroel 170, 08036 Barcelona, Spain; 5 Department of Medical Oncology, Hospital Universitario San Carlos, Dr Martín Lagos s/n, 28040 Madrid, Spain; 6 Department of Medical Oncology, Instituto Catalán de Oncología, Avda. Gran Vía Km. 2.7, 08907 L'Hospitalet de Llobregat, Barcelona, Spain; 7 Department of Medical Oncology, Instituto Valenciano de Oncología, Prof. Beltrán Báguena 19, 46009 Valencia, Spain; 8 Department of Medical Oncology, Hospital Trías y Pujol, Ctra. del Canyet s/n, 08916 Badalona, Spain; 9 Department of Medical Oncology, Hospital Miguel Servet, P° Isabel la Católica 1-3, 50009 Zaragoza, Spain

**Keywords:** doxorubicin, gemcitabine, soft tissue sarcoma, gemcitabine triphosphate

## Abstract

The aim of the study was to determine the dose-limiting toxicity and maximum tolerated dose of a first-line combination of doxorubicin and gemcitabine in adult patients with advanced soft tissue sarcomas and to explore its activity and toxicity, and the presence of possible interactions between these agents. Patients with measurable disease were initially treated with doxorubicin 60 mg m^−2^ by i.v. bolus on day 1 followed by gemcitabine at 800 mg m^−2^ over 80 min on days 1 and 8, every 21 days. Concentrations of gemcitabine and 2′,2′-difluorodeoxyuridine in plasma, and gemcitabine triphosphate levels in peripheral blood mononuclear cells were determined during 8 h after the start of gemcitabine infusion. Myelosuppression and stomatitis were limiting toxicities, and the initial dose level was applied for the Phase II trial, where grade 3–4 granulocytopenia occurred in 70% of patients, grade 3 stomatitis in 46% and febrile neutropenia in 20%. Objective activity in 36 patients was 22% (95% CI: 9–35%), and a 50% remission rate was noted in leiomyosarcomas. Administration of doxorubicin preceding gemcitabine significantly reduced the synthesis of gemcitabine triphosphate. Clinical activity, similar to that of single-agent doxorubicin, and the toxicity encountered do not justify further studies with this schedule of administration.

Doxorubicin (DXR) and ifosfamide are the only two drugs with consistent activity against advanced soft tissue sarcoma (ASTS) of the adult and constitute the backbone of combinations to treat this group of diseases. However, as overall efficacy of chemotherapy in ASTS is limited, new approaches are needed to improve therapeutic results (Brennan *et al*, 2001, p 1841). Among new agents, gemcitabine has shown some efficacy either as first-line or in those patients refractory to DXR and ifosfamide, with an activity that varies from 3 to 18% (Amodio *et al*, 1999; Merimsky *et al*, 2000; Spath-Schwalbe *et al*, 2000; Patel *et al*, 2001; Svancarova *et al*, 2002; Okuno *et al*, 2003). Therefore, we decided to explore the activity of a combination of DXR and gemcitabine.

Gemcitabine has to be phosphorylated to its diphosphate and triphosphate (dFdCTP) forms to exert its biologic effects, and it is deaminated to generate 2′,2′-difluorodeoxyuridine (dFdU), which would be devoid of antitumour activity (Plunkett *et al*, 1995). Intracellular accumulation of dFdCTP by peripheral blood mononuclear cells (PBMCs) is optimal when gemcitabine is delivered at a fixed dose-rate (FDR) infusion of 10 mg m^−2^ min^−1^ (Abbruzzese *et al*, 1991; Grunewald *et al*, 1991). The recommended dose for FDR infusion gemcitabine is 1500 mg m^−2^ for 3 out of 4 consecutive weeks (Brand *et al*, 1997; Touroutoglou *et al*, 1998). Gemcitabine and DXR had been combined to treat patients with advanced breast carcinoma in a study where both agents were given for 3 out of 4 consecutive weeks, with median tolerated doses of 800 mg m^−2^ for gemcitabine and 20 mg m^−2^ for DXR (Pérez-Manga *et al*, 2000). Because DXR with FDR infusion gemcitabine had not been given before, we performed a Phase I trial to determine the dose-limiting toxicity (DLT) and the recommended dose for subsequent studies. Initial doses were DXR 60 mg m^−2^ on day 1, followed by gemcitabine 800 mg m^−2^ on days 1 and 8. This study was followed by a Phase II trial to evaluate the activity and obtain additional information on the toxicity of this regimen. Another objective was to detect possible interactions between DXR and gemcitabine.

## MATERIALS AND METHODS

### Patients

Eligible patients should have an histologic diagnosis of ASTS, with local or advanced unresectable and measurable disease, no prior chemotherapy, performance status ⩽2 (WHO), adequate bone marrow (leucocytes ⩾3.0 × 10^9^ l^−1^, granulocytes ⩾1.5 × 10^9^ l^−1^, platelets ⩾100 × 10^9^ l^−1^), liver (bilirubin ⩽1.5-fold and AST and ALT <2.5-fold upper normal limits) and renal (creatinine ⩽1.5 mg dl^−1^) functions and left ventricular ejection fraction (LVEF) ⩾50%. Patients <18 years old, with severe associated diseases or active infection, CNS metastases, NYHA cardiac disease of grade >2 were excluded. Spanish Ministry of Health and Ethics Committee of all participating institutions approved the study, and informed patients signed a consent form.

### Treatment and study design

Patients received DXR and gemcitabine every 3 weeks. The dose level of DXR was fixed at 60 mg m^−2^ and dose levels for gemcitabine in the Phase I were 800 (dose level 1), 1000 (dose level 2) and 1200 mg m^−2^ (dose level 3). On day 1, DXR was delivered by i.v. bolus, immediately followed by gemcitabine (10 mg m^−2^ min^−1^), and on day 8 the dose of gemcitabine was repeated. Three new patients were entered at each dose level. If DLT was encountered in one of three patients, three additional patients were entered at that dose level, and if two patients had DLT at a particular dose level, this would represent the maximum tolerated dose (MTD), and the prior level would be applied in the Phase II study. Dose-limiting toxicity was the presence of febrile neutropenia, grade 4 neutropenia or thrombocytopenia lasting more than 4 days, grade 3 thrombocytopenia with bleeding, any grade 3–4 non-haematologic toxicity (except for nausea and vomiting) or cardiac toxicity ⩾ grade 2. Antiemesis consisted of anti-HT_3_ plus dexamethasone, with dexamethasone omitted in patients participating in the pharmacokinetic study.

### Toxicity

Toxicity was evaluated according to the NCI Common Toxicity Criteria, Version 1.0. Cycles were delivered on schedule if granulocytes ⩾1.5 × 10^9^ l^−1^ and platelets ⩾100 × 10^9^ l^−1^, and no grade 3–4 non-haematologic toxicity were present. In other situations, the treatment was delayed, and if no recovery had occurred within 3 weeks the patient was removed from the study. If a patient had DLT in the previous cycle or a 2-week delay was necessary, the dose of both DXR and gemcitabine was reduced by 25%. Patients with more than two dose reductions abandoned the study. On day 8, the dose of gemcitabine was reduced by 25% with granulocytes 500–1000 × 10^9^ l^−1^ and/or platelets 50–100 × 10^9^ l^−1^, and omitted if granulocytes <500 × 10^9^ l^−1^ and/or platelets <50 × 10^9^ l^−1^. Both DXR and gemcitabine doses were reduced by 25 or 50%, respectively, in the presence of any non-haematologic toxicity of grade 3 or 4. If any toxicity grade 3–4 recurred after a dose reduction, the patient was retired from the study. A maximum of six cycles were established per protocol.

### Study parameters and criteria of response

Patients were controlled weekly during the Phase I to check analytical and general toxicity. The LVEF was determined every other cycle during the Phase I study, and at the end of treatment in the Phase II study. During Phase II, analytical monitoring was performed on days 1, 8 and 21. All patients receiving at least one cycle of therapy were considered evaluable for toxicity. The first evaluation of activity was performed after two cycles or 6 weeks on study. Target lesions were measured every 6 weeks or whenever progressive disease was suspected, applying RECIST criteria for efficacy (Therasse *et al*, 2000), and objective remissions were externally reviewed. The duration of overall response was measured from the day it was first detected until the date of progression. Time to progression was the time elapsed from inclusion until detection of progressive disease. Progression-free rate was the proportion of patients without progression at a given time.

The dose intensity per patient was calculated by dividing total dose given (mg m^−2^) by the time elapsed from the first to the last dose plus 3 additional weeks. Relative dose intensity was the ratio of received to projected dose intensity.

### Pharmacokinetic analysis

On days 1 and 8 of the same cycle, blood samples were collected in heparinised tubes containing tetrahydrouridine. Samples were obtained at baseline, at 30 and 45 min, just before completion of infusion and hourly during 8 h. Samples were placed on ice, centrifuged and plasma stored at −26°C. Peripheral blood mononuclear cells were obtained through a Ficoll–Hypaque gradient, and isolated cells were preserved at −70°C. Gemcitabine and dFdU plasma concentrations and dFdCTP levels in PBMCs were determined by reverse-phase HPLC, according to published methods (Losa *et al*, 2004, 2005). The lower limits of quantification for gemcitabine and dFdCTP were, respectively, 0.36 and 0.174 *μ*g ml^−1^.

Gemcitabine concentration at steady state (*C*_ss_) was the mean of values after equilibrium was reached. *C*_max_ was the highest concentration detected for dFdU and dFdCTP. The area under the concentration–time curve (AUC_0−8 h_) for gemcitabine, dFdU and dFdCTP was obtained by applying the linear trapezoidal rule from time 0 until 8 h from start of gemcitabine infusion. AUC_inf_ was the accumulation of dFdCTP in PBMCs during gemcitabine infusion. Total body clearance (Cl) for gemcitabine was obtained from the relation dose/AUC. The half-life and the apparent volume of distribution of gemcitabine (*V*_d_=Cl/*k*, where *k* is the elimination constant for gemcitabine) were estimated only in patients with adequate infusional or postinfusional data.

Plasma concentrations of DXR and doxorubicinol (DOL) were determined according to a published method (Maessen *et al*, 1988) with slight modifications following the introduction of daunorubicin as an internal standard, which allowed us to fully validate the method. AUC_1−8 h_ of both DXR and DOL was estimated by applying the log-linear trapezoidal rule. These data, linearly corrected for a dose of 50 mg m^−2^, were compared with those of a series of 16 patients treated with DXR at 50 mg m^−2^ delivered by i.v. bolus, previously studied in our laboratory. In these historical controls, AUC_0−∞_ for DXR was 2392±556 nM h, Cl 62±15 l h^−1^ and terminal elimination half-life 35.7±11.5 h (mean±s.d.).

### Statistics

Sample size for the Phase II trial was calculated according to the two-stage optimal design of Simon (1989), with *α*=0.10, *β*=0.10, *P*_0_=20% and *P*_1_=40%. If less than three remissions occurred in the first 17 patients, the study should be interrupted because objective activity would be lower than 20%. If ⩽10 partial remissions were observed in 37 evaluable patients, the schedule would have an activity lower than 40%, and will not be considered for further development. The study continued after the positive results of the first step, but it was closed because only eight objective remissions were observed in 36 fully assessable patients.

Mean±s.d. of the different pharmacokinetic parameters was determined, and data on day 8 were pair-compared with those on day 1 by the Wilcoxon signed ranks test. The values for DXR and DOL were compared with historical data with the Student's *t*-test. All *P*-values presented are two-sided.

## RESULTS

### Phase I trial

From September 2001 to June 2002, 11 patients were included (Table 1) and the toxicity of the first cycle was considered to determine the MTD. Of the first three patients treated at dose level 1, one had haematologic DLT and died of septic shock after haematologic recovery, whereas another referred asthenia grade 2. In the next three patients treated at dose level 1, one episode of stomatitis grade 3 and one episode of grade 2 asthenia were observed. At this stage, with two out of six patients showing DLT and a rather low dose of gemcitabine, we decided to confirm that dose level 1 represented in fact the MTD. Therefore, three more patients were included at dose level 1 without observing further episodes of DLT. Then, dose Level 2 was explored, with two out of two patients presenting DLT. The first patient had grade 3 stomatitis and febrile neutropenia, without haematologic recovery by day 21; the second patient had grade 4 neutropenia lasting more than 7 days. The Phase II study was thus conducted at DXR 60 mg m^−2^ on day 1 and gemcitabine 800 mg m^−2^ on days 1 and 8, every 3 weeks. The toxicity of the 30 cycles (median 3, range 1–6) received by nine Phase I patients treated at the recommended dose is presented combined with that of Phase II patients. In nine patients assessable for efficacy, two partial remissions (one patient with a uterine leiomyosarcoma and one patient with an unclassified sarcoma of the limb), three stabilisations and four progressions were noted.

### Phase II trial

From July 2002 to December 2003, 40 patients were enrolled, and four ineligible patients (one adenocarcinoma, one extraskeletal myxoid chondrosarcoma and two gastrointestinal stromal tumours) were excluded from any analysis. Thirty-six patients (Table 1) were valid for activity and one was not assessable for haematologic toxicity. A total of 162 cycles were delivered with a median of 4.5 cycles (range 1–9) per patient. Two patients received only one cycle due to progressive disease.

### Toxicity

In Table 2, we present the haematologic toxicity observed in 192 cycles and 44 patients. The nadir and recovery of granulocytes occurred on days 13 (5–35) and 21 (14–33), respectively (mean, range). Packed red blood cells were required by 18 patients in 28 cycles, and platelets were transfused to one patient in one cycle. One patient presented a haemoglobin value of 6.8 g dl^−1^ after tumour bleeding. Non-haematologic toxicity is presented in Table 3.

Grade 2 increase of ALT values was noted in 9% of patients, and grade 2 and 3 increase in *γ*-glutamyl transferase values occurred in 9 and 4%, respectively. Alopecia was universal. Other toxic effects noted were fever grade 1 (four patients), erythema plus pruritus (two) or repeated episodes of conjunctivitis (one). Febrile neutropenia occurred in 10 patients and 20 cycles; only two of those episodes were noted among 14 patients receiving gemcitabine on day 8 with granulocytes 500–1000 × 10^9^ l^−1^. Sixteen patients in 21 cycles required admission to hospital for treatment-related side effects. Ten patients, including three Phase I patients, had excessive toxicity consisting of grade 3 stomatitis plus grade 3–4 haematologic toxicity (four), myelosuppression that required successive dose reductions (three), a decrease in LVEF to 45% (one), interstitial pneumonitis (one) or grade 3 asthenia plus severe infection (one), which led to premature interruption of therapy.

### Dose intensity

The dose of DXR was reduced in 27% of cycles, and that of gemcitabine in 40%, whereas 12% of cycles were delayed, usually owing to lack of haematologic recovery or persistent stomatitis. The dose of gemcitabine on day 1 was reduced in 32% of cycles, and that of day 8 in 35%, this dose being held in 8% of cycles. Median dose intensities of DXR and gemcitabine were, respectively, 18.2 mg m^−2^ week^−1^ (range 13–21) and 436 mg m^−2^ week^−1^ (range 243–562 mg m^−2^ week^−1^), with a relative dose intensity of 89±11% for DXR and 82±15% for gemcitabine (mean±s.d.).

### Response to therapy and clinical evolution

In 36 patients, one complete and seven partial remissions, 18 stabilisations and 10 progressions were noted (22% response rate; 95% CI: 9–35%). Sensitive histotypes were leiomyosarcoma (three out of six), fibrosarcoma (one out of two), sarcoma phyllodes (one out of one), malignant fibrous histiocytoma (one out of four), liposarcoma (one out of eight) and unclassified sarcoma (one out of five). Responding leiomyosarcomas originated in the trunk, uterus or retroperitoneum (one each). Duration of response was 30±21 weeks (mean±s.d.), median time to disease progression was 28 weeks (95% CI: 21–34 weeks) and the progression-free rate (±s.e.) at 3 and 6 months was 69±0.08 and 56±0.08%, respectively. Eventually, 27 patients left the study owing to progressive disease and nine retired prematurely: seven because of excessive toxicity and two to follow another therapeutic procedures. At analysis, with a median follow-up of 35 months, five patients were alive (two without evidence of disease), two were lost to follow-up with active disease and 29 had died of disease. Median overall survival was 60 weeks (95% CI: 39–81 weeks).

### Pharmacokinetics

Pharmacokinetic parameters of gemcitabine and dFdU in plasma (*n*=8), and data from dFdCTP accumulation in PBMCs (*n*=6) on days 1 and 8 are presented in Table 4. Gemcitabine half-life was 12.5±4.31 min (range 6.35–18 min) and its apparent volume of distribution was 76.35±44.2 l (range 24.30–156 l (mean±s.d.) (*n*=6)). Gemcitabine *C*_ss_ was usually reached at 30 min, and plasma dFdU peaked at 90.0±17.2 min from start of gemcitabine infusion. Gemcitabine parameters did not differ between days 1 and 8. AUC_0−8 h_ of dFdU was 21% lower on day 8 (gemcitabine alone) than on day 1 (DXR preceding gemcitabine) (*P*=0.02), and the relation AUC_0−8 h_ dFdU/AUC gemcitabine was higher on day 1 (*P*=0.01). With regard to dFdCTP concentration in PBMCs, *C*_max_ (*P*=0.03), AUC_inf_ (*P*=0.03) and AUC_0−8 h_ (*P*=0.04) were higher on day 8.

AUC_1−8 h_ for DXR and DOL (mean±s.d.) were 281±122 and 148±100 nM h, respectively, for gemcitabine patients, and 256±61 and 142±66 nM h for historical controls (*P*=0.60 for DXR and *P*=0.88 for DOL comparison).

## DISCUSSION

This is the first study designed in ASTS to evaluate the toxicity profile and the activity of DXR plus FDR infusion gemcitabine delivered as first-line therapy. In the Phase I study, dose-limiting stomatitis and neutropenia were noted in two of six patients treated at dose level 1 and, according to the study design, this represented the MTD and a lower dose level should have been opened. However, we decided to include three additional patients at dose level 1 to confirm our findings. It was considered that the dose of gemcitabine was already low when the recommended dose for FDR infusion was 1500 mg m^−2^ (weekly schedule) (Brand *et al*, 1997), and data from our group, subsequently published (Buesa *et al*, 2004), had shown that gemcitabine 1800 mg m^−2^ FDR plus DTIC 500 mg m^−2^, both given every 2 weeks, were well tolerated. Because none of those three new patients had DLT, dose level 2 was explored and the Phase I closed when two of two patients developed DLT at this dose level.

The Phase II was conducted at DXR 60 mg m^−2^ on day 1 with gemcitabine 800 mg m^−2^ infused over 80 min on days 1 and 8 (dose level 1), repeated every 3 weeks. This schedule was poorly tolerated, which contrasts with the toxicity of single-agent DXR administered at 75 mg m^−2^ every 3 weeks (Borden *et al*, 1990; Santoro *et al*, 1995). In three first-line studies conducted with a combination of gemcitabine followed by DXR in patients with different tumour types, projected dose intensities were 10–25 mg m^−2^ week^−1^ for DXR and 600–833 mg m^−2^ week^−1^ for gemcitabine. Authors communicate, in general, low levels of toxicity, with stomatitis greater than grade 2 in 14–22% of patients and grade 4 neutropenia in 14–21% (Pérez-Manga *et al*, 2000; Gómez *et al*, 2001; Yang *et al*, 2002). Exposure to 4-epi-doxorubicin followed by gemcitabine proved to be synergistic in cancer cell lines grown *in vitro*, with a 65% increase in DNA damage when compared with the reverse sequence or simultaneous administration (Zoli *et al*, 2004). According to this finding, one might hypothesise that a higher DNA damage combined with a tissue-specific sensitivity could explain the poor mucosal and haematologic tolerance of our schedule.

This trial was accompanied by an exploratory study of the pharmacokinetics of gemcitabine and of dFdCTP accumulation in PBMCs. The paired comparison of data obtained on day 1 (DXR preceding gemcitabine) and on day 8 (gemcitabine only) indicates that DXR interferes with gemcitabine activation to dFdCTP by PBMCs, and facilitates gemcitabine deamination (Table 4). The lower dFdCTP synthesis on day 1 would not be secondary to a decrease in gemcitabine membrane transport, due to the higher gemcitabine deamination observed on day 1 and that most of gemcitabine deamination occurs intracellularly, with plasma cytidine deaminase activity playing a limited role (Abbruzzese *et al*, 1991). It seems unlikely that the dose of gemcitabine given on day 1 would induce an increase in PBMC deoxycytidine kinase activity, as it has been detected in pancreatic cell lines *in vitro* (Giovannetti *et al*, 2004). The pharmacokinetics of DXR during the limited period studied was not affected by gemcitabine when compared with historical controls, similar to the results of other studies (Pérez-Manga *et al*, 2000; Fogli *et al*, 2002). The administration of gemcitabine followed by DXR would probably not influence gemcitabine activation and perhaps could offer a better therapeutic index than the present schedule.

The 22% remission rate detected (95% CI: 9–35%) is within the 14–25% range reported for single-agent first-line DXR delivered at 70–75 mg m^−2^ every 3 weeks (Brennan *et al*, 2001, p 1841). Apparently, the addition of gemcitabine did not increase DXR activity, although this could only be ascertained by a comparative study. Our results contrast with those of the combination of gemcitabine and docetaxel (TXT), an agent with limited activity in ASTS (van Hoesel *et al*, 1994; Verweij *et al*, 2000). This combination induced a 43–53% remission rate in two Phase II studies, an efficacy that was almost limited to patients with leiomyosarcoma of any origin (Hensley *et al*, 2002; Leu *et al*, 2004). In our series, 50% of leiomyosarcoma patients responded to therapy, which confirms the sensitivity of this histotype to gemcitabine-containing combinations (Buesa *et al*, 2004). In those studies, only gemcitabine was given on day 1, whereas on day 8 gemcitabine preceded TXT, a sequence that was synergistic in an osteosarcoma and a breast cancer cell line (Leu *et al*, 2004); however, in lung cancer cell lines, the opposite sequence was more effective (Zoli *et al*, 1999). Similarly, *in vitro* studies have shown that the effects of a combination of DXR and gemcitabine depend both on the sequence of administration and on the particular cell line studied (Chow *et al*, 2000; Zoli *et al*, 2004).

The administration of gemcitabine followed by DXR, or the delivery of DXR on day 8, would perhaps offer a better therapeutic index than the present schedule, which cannot be recommended for further study.

## Figures and Tables

**Table 1:** Patient characteristics

	**Phase I**	**Phase II**
Number	11	36
Male/female	5/6	20/16
Age (median, range)	51 (35–69)	59 (23–80)
		
*Performance status*		
0	3	7
1	4	25
2	4	4
		
*Histologic type of sarcoma*		
Liposarcoma	3	9
Leiomyosarcoma	2	6
Malignant fibrous histiocytoma	—	4
Angiosarcoma	1	2
Other or unclassified	5	15
		
*Grade of malignancy*		
1	1	7
2	2	6
3	8	23
		
*Primary site*		
Retroperitoneum	4	11
Trunk and limbs	2	15
Uterine	2	2
Other	3	8

**Table 2:** Haematologic toxicity (% of patients)

	**NCI CTC grade**				
	**0**	**1**	**2**	**3**	**4**
Haemoglobin	4	15	62	17	2
Leucocytes	4	7	36	40	13
Granulocytes	7	4	19	21	49
Platelets	53	9	23	13	2

**Table 3:** Non-haematologic toxicity (% of patients)

	**NCI CTC grade**				
	**0**	**1**	**2**	**3**	**4**
Nausea	35	35	28	2	—
Vomiting	49	30	19	2	—
Diarrhoea	64	13	19	4	—
Anorexia	49	28	23	—	—
Asthenia	11	23	53	13	—
Stomatitis	23	17	14	46	—
Oesophagitis	62	15	15	8	—
Cutaneous	81	11	8	—	—
Hepatic	92	6	—	2	—
Flu-like	85	11	4	—	—
Febrile neutropenia	—	—	—	14	6

**Table 4:** Pharmacokinetics of gemcitabine and its metabolites

	**Day 1**	**Day 8**	***P***
*Gemcitabine (n*=*8)*			
*C*_ss_ (*μ*M)	16±6.1	19.1±9.5	0.33
AUC (*μ*M h)	21.1±7.2	20.5±5.4	0.80
Cl (l min^−1^)	4.7±1.3	4.3±1.3	1.00
			
*dFdU (n*=*8)*			
*C*_max_ (*μ*M)	65.3±15.4	62.1±12.2	0.40
AUC_0–8 h_ (*μ*M h)	364±78	286±78	0.02
AUC_0–8 h_ dFdU/AUC gemcitabine	18.3±5.1	14.5±4.6	0.01
			
*dFdCTP (n*=*6)*			
*C*_max_ (pmol 10^−6^ PBMC)	177.3±117.4	236±113	0.02
AUC_inf_ (pmol h 10^−6^ PBMC)	81±39	141±64	0.03
AUC_0–8 h_ (pmol h 10^−6^ PBMC)	734±370	957±262	0.04

Patients were treated with doxorubicin (60 mg m^−2^) immediately followed by gemcitabine (800 mg m^−2^ over 80 min) on day 1, and only with gemcitabine (800 mg m^−2^ over 80 min) on day 8.

dFdU=difluorodeoxyuridine; dFdCTP=gemcitabine triphosphate in peripheral blood mononuclear cells (PBMCs); *C*_ss_=concentration at steady state; AUC_0–8h_=area under the curve for the first 8 h from start of gemcitabine infusion; Cl=total body clearance; *C*_max_=maximum concentration; AUC_inf_=dFdCTP accumulation in PBMCs during gemcitabine infusion. Values represent mean±s.d. *P*=*P*-value of the Wilcoxon sign test for paired comparisons.

## References

[bib1] Abbruzzese JL, Grunewald R, Weeks EA, Gravel D, Adams T, Nowak B, Mineishi S, Tarassoff P, Satterlee W, Raber MN (1991) A phase I clinical, plasma, and cellular pharmacology study of gemcitabine. J Clin Oncol 9: 491–498 1999720 10.1200/JCO.1991.9.3.491

[bib2] Amodio A, Carpano S, Manfredi C, Del Monte G, Di Lauro L, Gionfra T, Conti F, Paoletti G, Lopez M (1999) Gemcitabine in advanced stage soft tissue sarcoma: a phase II study. Clin Ther 150: 17–20 10367540

[bib3] Borden EC, Amato DA, Edmonson JH, Ritch PS, Shiraki M (1990) Randomized comparison of doxorubicin and vindesine to doxorubicin for patients with metastatic soft-tissue sarcomas. Cancer 66: 862–867 2201431 10.1002/1097-0142(19900901)66:5<862::aid-cncr2820660509>3.0.co;2-r

[bib4] Brand R, Capadano M, Tempero M (1997) A phase I trial of weekly gemcitabine administered as a prolonged infusion in patients with pancreatic cancer and other solid tumors. Invest New Drugs 15: 331–341. DOI: 10.1023/A:100598117532 9547676 10.1023/a:1005981317532

[bib5] Brennan MF, Alektiar KM, Maki RG (2001) Sarcomas of the soft- tissue and bone. In Cancer: Principles and Practice of Oncology, DeVita VT, Hellman S, Rosenberg SA (eds) pp 1841–1891. Philadelphia: Lippincott Williams and Wilkins

[bib6] Buesa JM, Losa R, Fernández A, Sierra M, Esteban E, Díaz A, López-Pousa A, Fra J (2004) Phase I clinical trial of fixed-dose rate infusional gemcitabine and dacarbazine in patients with advanced soft tissue sarcomas with assessment of gemcitabine triphosphate accumulation. Cancer 101: 2261–2269. DOI: 10.1002/cncr.20612 15484216 10.1002/cncr.20612

[bib7] Chow KU, Ries J, Weidmann E, Pourebrahim F, Napieralski S, Stieler M, Boehrer S, Rummel MJ, Stein J, Hoelzer D, Mitrou PS (2000) Induction of apoptosis using 2′,2′,difluorodeoxycytidine (gemcitabine) in combination with antimetabolites or anthracyclines on malignant lymphatic and myeloid cells. Antagonism or synergism depends on incubation schedule and origin of neoplastic cells. Ann Hematol 79: 485–492. DOI: 10.1007/s00270000181 11043419 10.1007/s002770000181

[bib8] Fogli S, Danesi R, Gennari A, Donati S, Conte PF, Del Tacca M (2002) Gemcitabine, epirubicin and paclitaxel: pharmacokinetic and pharmacodynamic interactions in advanced breast cancer. Ann Oncol 13: 919–927. DOI: 10.1093/annonc/mdf164 12123338 10.1093/annonc/mdf164

[bib9] Giovannetti E, Mey V, Danesi R, Mosca I, Del Tacca M (2004) Synergistic cytotoxicity and pharmacogenetics of gemcitabine and pemetrexed combination in pancreatic cancer cell lines. Clin Cancer Res 10: 2936–2943 15131028 10.1158/1078-0432.ccr-03-0520

[bib10] Gómez H, Kahatt C, Falcon S, Santillana S, Hurtado de Mendoza F, Valdivia S, Vallejos C, Otero J, Pen DLK (2001) A phase II study of neoadjuvant gemcitabine plus doxorubicin in stage IIIB breast cancer: a preliminary report. Semin Oncol 28(Suppl 10): 57–61. DOI: 10.1053/sonc.2001.22532 11510035 10.1053/sonc.2001.22532

[bib11] Grunewald R, Abbruzzese JL, Tarasoff P, Plunkett W (1991) Saturation or 2′,2′-difluorodeoxycytidine 5′-triphosphate accumulation by mononuclear cells during a phase I trial of gemcitabine. Cancer Chemother Pharmacol 27: 258–262. DOI: 10.1007/BF00685109 1998982 10.1007/BF00685109

[bib12] Hensley ML, Maki R, Venkatraman E, Geller G, Lovegren M, Aghajanian C, Sabbatini P, Tong W, Barakat R, Spriggs DR (2002) Gemcitabine and docetaxel in patients with unresectable leiomyosarcoma: results of a phase II trial. J Clin Oncol 20: 2824–2831. DOI: 10.1200/JCO.2002.11.050 12065559 10.1200/JCO.2002.11.050

[bib13] Leu KM, Ostruszka LJ, Shewach D, Zalupski M, Sondak V, Biermann JS, Lee JS, Couwlier C, Palazzolo K, Baker LH (2004) Laboratory and clinical evidence of synergistic cytotoxicity of sequential treatment with gemcitabine followed by docetaxel in the treatment of sarcoma. J Clin Oncol 22: 1706–1712. DOI: 10.1200/JCO.2004.08.043 15117993 10.1200/JCO.2004.08.043

[bib14] Losa R, Siera MI, Blay P, Blanco D, Buesa JM (2004) Determination of gemcitabine triphosphate in peripheral blood mononuclear cells by reverse-phase HPLC. Chromatographia 59: 493–496. DOI: 10.1365/s10337-004-0210-3

[bib15] Losa R, Sierra MI, Guardado C, Fernández A, Gión MO, Blanco D, Buesa JM (2005) Development and validation of an ion pair HPLC method for gemcitabine and 2′,2′-difluoro-2′-deoxyuridine determination. Anal Chim Acta 528: 255–260. DOI: 10.1016/j.aca.2004.10.032

[bib16] Maessen PA, Pinedo HM, Mross KB, van der Vijgh JF (1988) New method for the determination of doxorubicin, 4′-epidoxorubicin and all known metabolites in cardiac tissue. J Chromatogr 424: 103–110 3163337 10.1016/s0378-4347(00)81080-4

[bib17] Merimsky O, Meller I, Flusser G, Kollender Y, Issakov J, Weil-Ben-Arush M, Fenig E, Neuman G, Sapir D, Ariad S, Inbar M (2000) Gemcitabine in soft tissue or bone sarcoma resistant to standard chemotherapy: a phase II study. Cancer Chemother Pharmacol 45: 177–181. DOI: 10.1007/s002800050027 10663634 10.1007/s002800050027

[bib18] Okuno S, Ryan LM, Edmonson JH, Priebat DA, Blum RH (2003) Phase II trial of gemcitabine in patients with advanced sarcomas (E1797). A trial of the Eastern Cooperative Oncology Group. Cancer 97: 1969–1973. DOI: 10.1002/cncr.11290 12673725 10.1002/cncr.11290

[bib19] Patel SR, Gandhi V, Jenkins J, Papadopolous N, Burgess MA, Plager C, Plunkett W, Benjamin RS (2001) Phase II clinical investigation of gemcitabine in advanced soft tissue sarcomas and window evaluation of dose rate on gemcitabine triphosphate accumulation. J Clin Oncol 19: 3483–3489 11481354 10.1200/JCO.2001.19.15.3483

[bib20] Pérez-Manga G, Lluch A, Alba E, Moreno-Nogueira JA, Palomero M, García-Conde J, Khayat D, Rivelles N (2000) Gemcitabine in combination with doxorubicin in advanced breast cancer: final results of a phase II pharmacokinetic trial. J Clin Oncol 18: 2545–2552 10893285 10.1200/JCO.2000.18.13.2545

[bib21] Plunkett W, Huang P, Xu YZ, Heinemann V, Grunewald R, Gandhi V (1995) Gemcitabine: metabolism, mechanisms of action and self-potentiation. Semin Oncol 22: 3–10 7481842

[bib22] Santoro A, Tursz T, Mouridsen H, Verweij J, Steward W, Somers R, Buesa J, Casali P, Spooner D, Rankin E, Kirkpatrick A, van Glabbeke M, van Oosterom A (1995) Doxorubicin *versus* CYVADIC *versus* doxorubicin plus ifosfamide in first-line treatment of advanced soft-tissue sarcomas: a randomized study of the EORTC Soft Tissue and Bone Sarcoma Group. J Clin Oncol 13: 1537–1545 7602342 10.1200/JCO.1995.13.7.1537

[bib23] Simon R (1989) Optimal two-stage designs for phase II clinical trials. Control Clin Trials 10: 1–10 2702835 10.1016/0197-2456(89)90015-9

[bib24] Spath-Schwalbe E, Genvresse I, Koschuth A, Dietzmann A, Grunewald R, Possinger K (2000) Phase II trial of gemcitabine in patients with pretreated advanced soft tissue sarcomas. Anticancer Drugs 11: 325–329 10912948 10.1097/00001813-200006000-00002

[bib25] Svancarova L, Blay JY, Judson IR, van Hoesel QG, van Oosterom AT, Le Cesne A, Keizer HJ, Hermans C, van Glabbeke M, Verweij J, Hogendoorn PC, Nielsen OS (2002) Gemcitabine in advanced adult soft-tissue sarcomas. A phase II study of the EORTC Soft Tissue and Bone Sarcoma Group. Eur J Cancer 38: 556–559. DOI: 10.1016/S0959-8049(01)00408-7 11872349 10.1016/s0959-8049(01)00408-7

[bib26] Therasse P, Arbuck SG, Eisenhauer EA, Wanders J, Kaplan RS, Rubinstein L, Verweij J, van Glabbeke M, van Oosterom AT, Christian MC, Gwyther SG (2000) New guidelines to evaluate the response to treatment in solid tumors. J Natl Cancer Inst 92: 205–216 10655437 10.1093/jnci/92.3.205

[bib27] Touroutoglou N, Gravel M, Raber N, Plunkett W, Abbruzzese JL (1998) Clinical results of a pharmacodynamically-based strategy for higher dosing of gemcitabine in patients with solid tumors. Ann Oncol 9: 1003–1008. DOI: 10.1023/A:1008487932384 9818075 10.1023/A:1008487932384

[bib28] van Hoesel QG, Verweij J, Catimel G, Clavel M, Kerbrat P, van Oosterom AT, Kerger J, Tursz T, van Glabbeke M, van Pottelsberghe C (1994) Phase II study with docetaxel (Taxotere) in advanced soft tissue sarcomas of the adult: EORTC Soft Tissue and Bone Sarcoma Group. Ann Oncol 5: 539–542 7918126 10.1093/oxfordjournals.annonc.a058909

[bib29] Verweij J, Lee SM, Ruka W, Buesa J, Coleman R, van Hoessel R, Seynaeve C, di Paola ED, van Glabbeke M, Tonelli D, Judson IR (2000) Randomized phase II study of docetaxel *versus* doxorubicin in first- and second-line chemotherapy for locally advanced or metastatic soft tissue sarcomas in adults: a study of the European Organization for Research and Treatment of Cancer Soft Tissue and Bone Sarcoma Group. J Clin Oncol 18: 2081–2086 10811673 10.1200/JCO.2000.18.10.2081

[bib30] Yang TS, Wang CH, Hsieh RK, Chen JS, Fung MC (2002) Gemcitabine and doxorubicin for the treatment of patients with advanced hepatocellular carcinoma: a phase I–II trial. Ann Oncol 13: 1771–1778. DOI: 10.1093/annonc/mdf303 12419750 10.1093/annonc/mdf303

[bib31] Zoli W, Ricotti L, Dal Susino M, Barzanti F, Frassineti GL, Folli S, Tesei A, Bacci F, Amadori D (1999) Docetaxel and gemcitabine activity in NSCLC cell lines and in primary cultures from human lung cancer. Br J Cancer 81: 609–615. DOI: 10.1038/sj.bjc.6690737 10574245 10.1038/sj.bjc.6690737 PMC2362882

[bib32] Zoli W, Ricotti L, Tesei A, Ulivi P, Campani AG, Fabbri F, Gunelli R, Frassineti GL, Amadori D (2004) Schedule-dependent cytotoxic interaction between epidoxorubicin and gemcitabine in human bladder cancer cells *in vitro*. Clin Cancer Res 10: 1500–1507 14977854 10.1158/1078-0432.ccr-1107-03

